# Comparing burden of ischemic stroke caused by high low-density lipoprotein cholesterol in global and China: trends and projections

**DOI:** 10.3389/fneur.2025.1622361

**Published:** 2025-08-13

**Authors:** Jiali Zhang, Mingxiu Liu, Junlin Yue, Liang Zuo, Qiuzhu Xu, Jie Yang, Gao Liu, Enli Cai

**Affiliations:** ^1^The Third Affiliated Hospital of Yunnan University of Chinese Medicine, Kunming Municipal Hospital of Traditional Chinese Medicine, Kunming, Yunnan, China; ^2^Bishan Hospital of Chongqing Medical University, Chongqing, China; ^3^The Second Affiliated Hospital of Yunnan University of Traditional Chinese Medicine, Kunming, Yunnan, China; ^4^Haikou Orthopedic and Diabetes Hospital of Shanghai Sixth People's Hospital, Haikou, Hainan, China; ^5^Yunnan University of Chinese Medicine, Kunming, Yunnan, China

**Keywords:** ischemic stroke, high low-density lipoprotein cholesterol, disease burden, risk factors, prediction

## Abstract

**Background:**

Ischemic stroke (IS) remains a major global cause of death and disability, with elevated low-density lipoprotein cholesterol (LDL-C) identified as a key modifiable risk factor. This study evaluates the global and Chinese burden of IS attributable to high LDL-C from 1990 to 2021, and projects future trends through 2046.

**Materials and methods:**

Data on IS mortality, disability-adjusted life years (DALYs), and age-standardized rates (ASRs) were sourced from the Global Burden of Disease (GBD) 2021 database. Analyses were stratified by age and sex. Temporal trends were assessed using Joinpoint regression, calculating the annual percentage change (APC) and the average annual percentage change (AAPC). Future burden was projected using the Nordpred model.

**Results:**

In 2021, IS deaths due to high LDL-C reached 936,192 globally (an increase of 43.04%) and 300,053 in China (an increase of 161.86%), despite age-standardized mortality rate (ASMR) declining by 43.16% and 12.61%, respectively. DALYs increased by 44.55% globally and 126.94% in China, with smaller reductions in age-standardized DALY rate (ASDR). Mortality and DALYs increased with age and were higher in men. China's elderly (≥90 years) exhibited particularly high mortality (1,058.69/100,000) compared to the global ≥95 age group (482.32/100,000). AAPCs indicated more consistent declines globally than in China. Projections estimate IS deaths will reach 1.86 million globally and 630,000 in China by 2046, with DALYs rising to 36.8 million and 11.4 million, respectively. China's projected ASMR and ASDR will remain higher than global averages.

**Conclusion:**

Despite declines in age-standardized rates, the absolute burden of IS attributable to high LDL-C continues to rise globally, driven by population aging and growth. China, in particular, faces a disproportionately higher burden compared to global trends, with slower progress in reducing these rates. Significant age- and sex-based disparities remain. These findings underscore the urgent need for targeted public health strategies to address the growing burden of IS, particularly in China, by focusing on effective LDL-C management.

## 1 Introduction

IS, also known as cerebral infarction, represents a common cerebrovascular disease primarily caused by thrombosis obstructing cerebral blood vessels, leading to ischemia, hypoxia, and subsequent necrosis of brain tissue (1, 2). It is the third leading cause of death globally, accounting for 10.70% of total mortality, and is also the fourth leading cause of DALYs (3). IS constitutes ~65.30% of all stroke cases, associated with high incidence, mortality, and disability rates, posing a significant public health challenge (4).

The GBD 2021 report indicates that the incidence of IS has significantly increased. From 1990 to 2021, the number of IS cases increased by 87.97%, the number of deaths rose by 55.00%, and DALYs increased by 52.37%. By 2021, there were 7.80 million cases, 3.59 million deaths, and 704 million DALYs (5). In China, stroke-related deaths account for more than a third of the global total, posing a major public health challenge. IS accounts for 86.80% of all stroke cases in China (6). In 2021, the death toll and DALYs from IS in China reached 1.18 million and 23.43 million, respectively, which are 2.75 and 2.36 times the levels recorded in 1990 (7). These findings demonstrate that IS remains the leading cause of DALYs in China. Despite improvements in the overall accessibility of healthcare, the continued aging of the population and other risk factors have resulted in a persistently high burden of stroke, creating substantial economic burdens on patients' families, healthcare systems, and society (8).

LDL-C is closely associated with the risk of IS. Studies have shown that reducing LDL-C levels can effectively decrease the incidence and recurrence of IS, particularly in high-risk populations (9). Among patients with IS, those with an LDL-C level target of <70 mg/dL have a significantly lower risk of subsequent cardiovascular events compared to those whose target range is between 90 mg/dL and 110 mg/dL (10). Despite widespread statin use in lipid-lowering therapy, some patients struggle to meet targets, necessitating additional medications like Ezetimibe or PCSK9 inhibitors to further reduce LDL-C levels (11). However, in certain high-risk patients, existing pharmacologic treatments are insufficient to lower LDL-C to the recommended levels, prompting exploration of new therapeutic strategies (12). Recent research indicates that the relationship between LDL-C levels and all-cause mortality after stroke follows a U-shaped curve, with both excessively low and high LDL-C levels in the acute phase of IS potentially increasing mortality risk (13). These findings indicate the need for treatment strategies that balance lipid-lowering efficacy with individualized risk profiles. Therefore, optimizing LDL-C control targets, especially in high-risk populations with a history of cardiovascular disease, remains a crucial pathway for preventing IS.

While LDL-C control is critical for the prevention of cardiovascular diseases, with the improvement of China's economic level and changes in dietary patterns, diets rich in high energy and fats have become more common. In addition, the decline in physical activity levels and increased life stress have contributed to a continuous rise in LDL-C levels (14). Recent evidence indicates a suboptimal achievement of LDL-C targets among patients with atherosclerotic cardiovascular disease (ASCVD) in China, with only 26.60% of high-risk individuals attaining recommended LDL-C levels, and an even lower attainment rate observed among those at very high risk (15). The 2021 GBD stroke analysis indicated that in East Asia, elevated high LDL-C ranks as the third most significant risk factor for IS, following high systolic blood pressure and air pollution from particulate matter (3). Research on the burden of stroke in China and its associated risk factors emphasizes that, beyond the shared risk factors for all stroke types (such as high systolic blood pressure, particulate matter pollution, and excessive sodium consumption), high LDL-C is identified as the most potent risk factor for IS (16).

Although previous studies (3, 17–19) have explored the global and Chinese burden of IS attributable to high levels of LDL-C and its associated risk factors, long-term trend analyses of the IS burden in China due to high LDL-C, as well as comparisons with global trends, remain limited. Furthermore, there is a lack of research on predicting future trends. This study uses data from the GBD project to analyze and compare the burden of IS attributable to high LDL-C in China and globally from 1990 to 2021. The study aims to provide insights for developing health policies and targeted primary prevention strategies for IS.

## 2 Materials and methods

### 2.1 Study design and data sources

We obtained data from the GBD 2021 database (http://ghdx.healthdata.org/gbd-results-tool). GBD 2021 is the most comprehensive global dataset on disease burden currently available. It evaluates disease burden using updated epidemiological data and improved methodologies, providing detailed data on health losses caused by 371 diseases, injuries, and disabilities, as well as 88 risk factors across 204 countries and regions (20, 21). In this database, IS is classified under the fourth-level etiology, representing the most specific categorization. We extracted data on IS attributable to high LDL cholesterol worldwide and in China from 1990 to 2021, including mortality, and DALYs estimates are presented in absolute numbers and as ASR per 100,000 population [with 95% Uncertainty Interval (UI)], stratified by age, sex. ASR were calculated using the global age-standardized population developed for the GBD study. These data provide detailed, age- and sex-specific information, which is used to analyze the impact of high LDL cholesterol on the burden of IS and serve as the basis for projections of future trends. The study adheres to the Guidelines for Accurate and Transparent Health Estimates Reporting (GATHER) (22).

### 2.2 Definitions

This study focuses on IS as a disease and high LDL-C as a major metabolic risk factor. According to the GBD 2021 definition, high LDL-C refers to a situation where the level of LDL-C in the blood exceeds the theoretical minimum risk exposure value of 1.30 mmol/L (50 mg/dL) (23). IS, as defined by WHO, is a neurological dysfunction caused by focal cerebral, spinal, or retinal infarction (24).

### 2.3 Statistical analysis

#### 2.3.1 Descriptive analysis

We performed descriptive analyses on IS-related indicators, including mortality rate and DALYs, attributable to high LDL-C from 1990 to 2021, both Globally and in China. It examines the distribution of these indicators across different genders and age groups. Additionally, the absolute values and ASR of IS mortality and DALYs were calculated, followed by an analysis and comparison of the trends in ASMR and ASDR between the two regions across different time periods. Potential regional and gender differences were also identified.

#### 2.3.2 Joinpoint regression analysis

Joinpoint regression analysis, a statistical method employing a log-linear regression framework, is primarily used to evaluate indicators' trend changes over time. In this study, we performed using the Joinpoint Regression Program (Version 5.3.0.0, National Cancer Institute) with log-linear model with a 95% confidence interval, allowing up to five potential Joinpoints. Joinpoints were identified using a grid search algorithm, and the optimal number of Joinpoints was determined through Monte Carlo permutation tests to detect significant trend changes (25). This model was used to compare the temporal trends of IS burden due to high LDL-C in China and globally, and to identify significant change points in these trends. APC and AAPC were calculated to analyze the trends and significance of the ASMR and ASDR. APC measures the percentage change in a variable over a specific time period, while AAPC reflects the average annual change of the variable over the entire study period. Unlike APC, which focuses on short-term fluctuations, AAPC emphasizes the overall trend over time, thereby offering more reliable guidance for long-term trend assessment. Statistical significance was assessed by comparing AAPC to zero, with a *P*-value of < 0.05 indicating statistical significance (25, 26).

#### 2.3.3 Predictive analysis

Based on historical data from 1990 to 2021, the Nordpred model was used to project the global and Chinese trends of IS mortality and DALYs attributable to high LDL-C from 2022 to 2046. The model operated under the default Poisson age-period-cohort framework with 5-year age groups and a power-5 link function. Key assumptions included: (i) incidence or mortality rates are proportional to age structure and population size; (ii) linear temporal trends within each 5-year period; and (iii) population projections aligned with the UN World Population Prospects (27). As a widely utilized tool for forecasting disease burden, the Nordpred model integrates temporal trends and demographic shifts to produce reasonable future estimates (28, 29). All analyses and visualizations were performed using R software (version 4.4.1, R Foundation for Statistical Computing, Vienna, Austria).

## 3 Results

### 3.1 Analysis of IS deaths and DALYs attributable to high LDL-C in the world and China

In 1990, the global number of deaths due to IS attributable to high LDL-C was 654,503 (95% UI: 210,161–1,103,893), with an ASMR of 20.03 (95% UI: 6.28–34.56) per 100,000. By 2021, these figures had increased to 936,192 (95% UI: 299,535–1,614,294) cases, representing a 43.04% rise in number and a decrease of 43.16% in the ASMR per 100,000, which was 11.38 (95% UI: 3.62–19.65). Additionally, the global DALYs due to high LDL-C-related IS in 1990 stood at 14,524,190 (95% UI: 5,187,367–23,068,198), with an ASDR of 390.89 (95% UI: 133.81–632.33) per 100,000. In 2021, DALYs increased to 20,977,424 (95% UI: 7,409,044–34,664,410), with a percentage change of 44.55% and an ASDR of 246.42 (95% UI: 86.47–406.28), reflecting a 36.96% decline in the rate per 100,000 (Table 1).

**Table 1:** Comparison of global and China deaths and DALYs due to high LDL-C-related IS in 1990 and 2021.

**Location**	**Metric**	**Number**		**Percentage change**	**Per 100,000**		**Percentage change**
		**1990**	**2021**		**1990**	**2021**	
Global (95% UI)	Death cases	654,503 (210,161–1,103,893)	936,192 (299,535–1,614,294)	43.04%	20.03 (6.28–34.56)	11.38 (3.62–19.65)	−43.16%
	DALYs	14,512,490 (5,187,367–23,068,198)	20,977,424 (7,409,044–34,664,410)	44.55%	390.89 (133.81–632.33)	246.42 (86.47–406.28)	−36.96%
China (95% UI)	Death cases	114,584 (37,396–197,386)	300,053 (92,516–527,457)	161.86%	18.23 (5.52–33.43)	15.93 (4.83–28.08)	−12.61%
	DALYs	3,018,680 (1,065,097–4,938,870)	6,850,566 (2,313,204–11,418,167)	126.94%	385.65 (129.83–647.51)	335.59 (112.75–566.25)	−12.98%

DALYs, disability-adjusted life years; LDL-C, low-density lipoprotein cholesterol; IS, Ischemic Stroke; UI, uncertainty intervals.

In China, the number of deaths attributed to high LDL-C-related IS was 114,584 (95% UI: 37,396–197,386) in 1990, with an ASMR of 18.23 (95% UI: 5.52–33.43) per 100,000. By 2021, the number of deaths rose to 300,053 (95% UI: 92,516–527,457), marking a 161.86% increase. The ASMR per 100,000 decreased by 12.61% to 15.93 (95% UI: 4.83–28.08). Similarly, for DALYs, China's 1990 figures were 3,018,680 (95% UI: 10,650,097–49,388,707), with an ASDR of 385.65 (95% UI: 129.83–647.51) per 100,000. By 2021, the DALYs attributed to high LDL-C-related IS increased to 6,850,566 (95% UI: 23,132,104–114,181,671), which represents a 126.94% increase. The ASDR per 100,000 in 2021 was 335.59 (95% UI: 112.75–566.25), a 12.98% decrease compared to 1990 (Table 1).

### 3.2 Trend analysis of the burden of IS attributable to high LDL-C in global and China from 1990 to 2021

**Figure 1:**
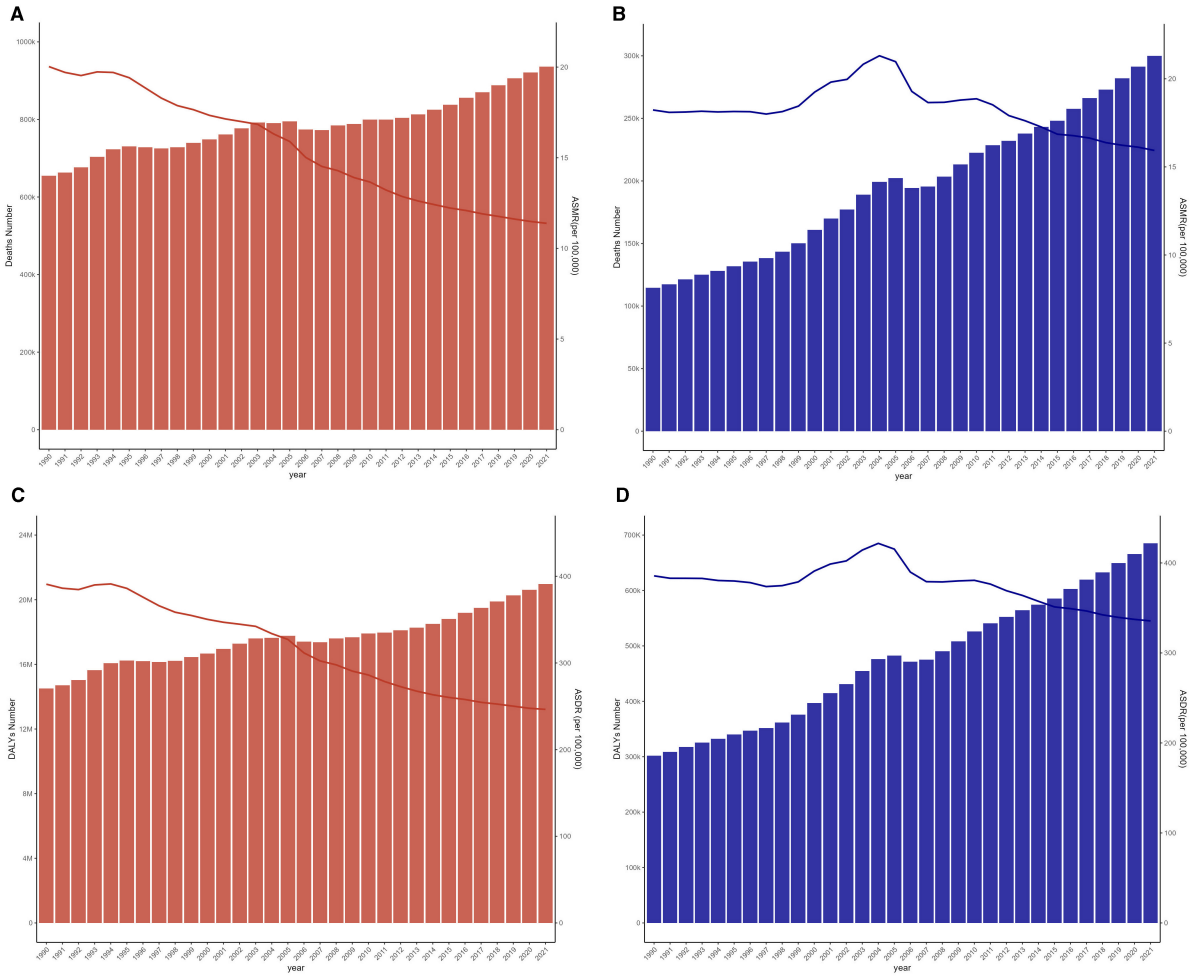
Changes in the disease burden of IS caused by high LDL-C in global and China from 1990 to 2021. **(A)** Trends in the number of deaths and ASMR in global from IS due to high LDL-C. **(B)** Trends in the number of deaths and ASMR in China from IS due to high LDL-C. **(C)** Trends in the DALYs and ASDR in global from IS due to high LDL-C. **(D)** Trends in the DALYs and ASDR in China from IS due to high LDL-C.

In 2021, the global number of deaths due to IS caused by high LDL-C was 936,192 (95% UI: 299,535–1,614,294), an increase of about 280,000 compared to 654,503 (95% UI: 210,161–1,103,893) in 1990. The ASMR decreased from 20.03 (95% UI: 6.28–34.56) per 100,000 to 11.38 (95% UI: 3.62–19.65) per 100,000, showing a 43.16% reduction (Figure 1A). In China, the number of deaths due to IS caused by high LDL-C was 300,053 (95% UI: 92,516–527,457) in 2021, an increase of nearly 190,000 deaths compared to 114,584 (95% UI: 37,396–197,386) deaths in 1990. The ASMR increased from 18.23 (95% UI: 5.52–33.43) per 100,000 in 1990, peaked in 2004, and then gradually declined to 15.93 (95% UI: 4.83–28.08) per 100,000 in 2021, marking a decrease of ~12.61% (Figure 1B).

Globally, the DALYs for IS due to high LDL-C increased from 14,512,490 (95% UI: 5,187,367–23,068,198) years in 1990 to 20,977,424 (95% UI: 7,409,044–34,664,410) years in 2021, representing a 44.55% increase. However, the ASDR declined from 390.89 (95% UI: 133.81–632.33) per 100,000 in 1990 to 246.42 (95% UI: 86.47–406.28) per 100,000 in 2021 (Figure 1C). In China, DALYs for IS due to high LDL-C increased from 3,018,680 (95% UI: 1,065,097–4,938,870) years in 1990 to 6,850,566 (95% UI: 2,313,204–11,418,167) years in 2021, representing a 126.94% increase. However, the ASDR per 100,000 population showed an increasing trend from 385.65 (95% UI: 129.83–647.51) in 1990, peaking in 2004, and subsequently declining to 335.59 (95% UI: 112.75–566.25) in 2021, marking a reduction of 12.98% (Figure 1D).

### 3.3 Burden of IS attributable to high LDL-C in the world and China in 2021 by age and gender

In 2021, the global mortality rate from IS attributable to high LDL-C exhibited clear age- and gender-specific trends. For males, the mortality rate was close to zero in the 25–29 years age group, while the rate was highest in the ≥95 years age group, reaching 482.32 (95% UI: 125.77–889.30) per 100,000. For females, the mortality rate also increased with age, but remained lower than that of males. Notably, in the ≥95 years age group, the female mortality rate surpassed that of males, reaching 646.67 (95% UI: 178.90–1,179.52) per 100,000.

**Figure 2:**
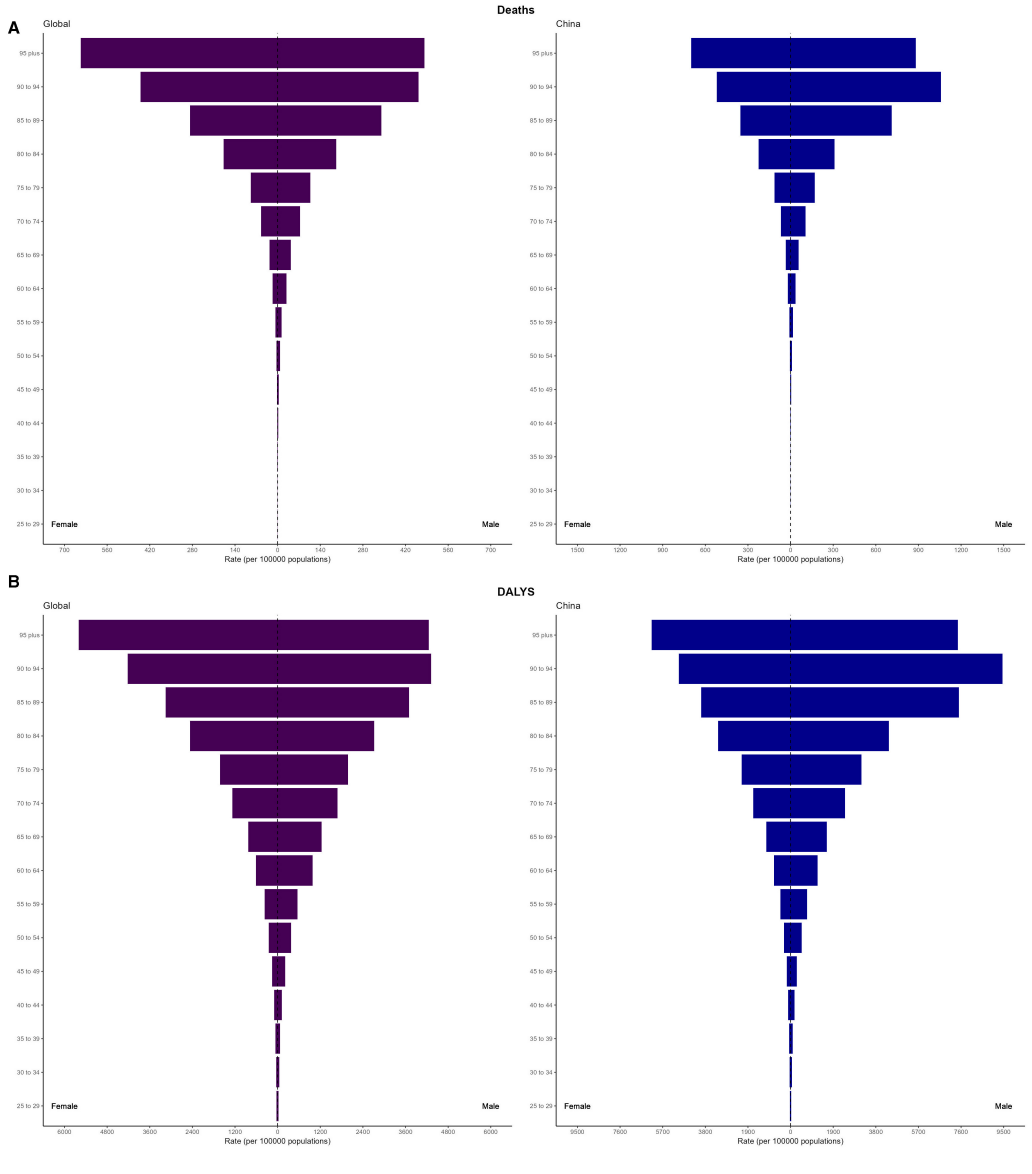
Age and sex-specific distribution of mortality rates and DALY rates due to high LDL-C-related IS in 2021 Globally and in China. **(A)** Mortality rates globally and in China. **(B)** DALY rates globally and in China.

In China, the mortality rate from IS attributable to high LDL-C in 2021 followed a similar trend but was higher than the global average. For males, the mortality rate gradually increased with age, with the highest rate observed in the 90–94 age group, 1,058.69 (95% UI: 268.82–2,019.77) per 100,000, notably exceeding that of the ≥95 age group, 881.59 (95% UI: 234.24–1,633.92) per 100,000. For females, the mortality rate also rose with age, mirroring the trend observed in males. Overall, the mortality rate for females was lower than for males; however, the gap gradually narrowed with increasing age. In the ≥95 years age group, the female mortality rate was the highest, reaching 699.15 (95% UI: 193.076–1,335.03) per 100,000. These results demonstrate, with increasing age, the mortality burden of IS in China is higher than the global average, particularly in the elderly population (Figure 2A).

In 2021, the DALY rate for IS attributable to high LDL-C also exhibited distinct age- and sex-specific patterns. For males, the DALY rate increased with age, with the highest rate observed in the 90–94 age group, 4,318.47 (95% UI: 1,098.67–7,974.55) per 100,000, which exceeded that of the ≥95 age group, 4,255.08 (95% UI: 1,093.64–7,801.04) per 100,000. For females, the DALY rate also rose with age, with the highest rate in the ≥95 age group, 5,599.26 (95% UI: 1,527.43–10,248.33) per 100,000. Except for the 25–34 and ≥95 age groups, males consistently exhibited higher DALY rates than females.

In China, the DALY rate for IS due to high LDL-C in 2021 also showed a marked age- and sex-specific trend. For males, the DALY rate increased with age, with the highest rate observed in the 90–94 age group, 9,445.25 (95% UI: 2,399.05–17,930.53) per 100,000, surpassing that of the ≥95 age group, 7,459.38 (95% UI: 1,976.82–13,834.08) per 100,000. For females, the DALY rate also increased with age, with the highest rate in the ≥95 age group, 6,196.06 (95% UI: 1,681.77–11,775.60) per 100,000. Although the rate for females was lower than that for males in this age group, it was still significantly higher than in other age groups (Figure 2B).

### 3.4 Jointpoint regression analysis of the burden of IS attributable to high LDL-C in the world and China

**Figure 3:**
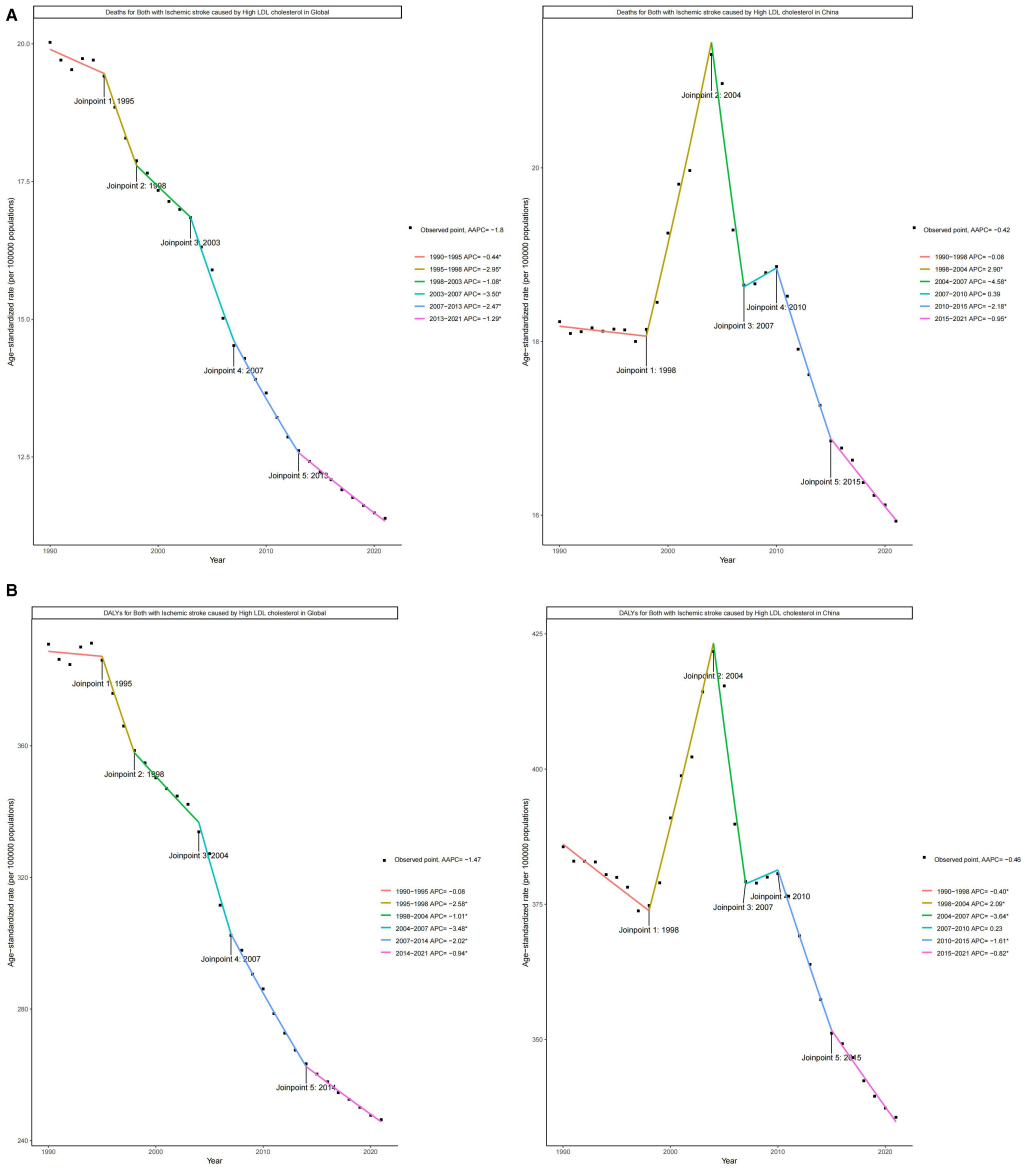
Joinpoint regression analysis of ASMR and ASDR for IS due to high LDL-C Globally and in China (1990–2021). **(A)** Joinpoint regression analysis of ASMR Globally and in China. **(B)** Joinpoint regression analysis of ASDR Globally and in China.

Figure 3 and Supplementary Table 1 present the Joinpoint regression analysis results of ASMR and ASDR for IS attributable to high LDL-C from 1990 to 2021. This analysis reveals distinct phases of change in the trends of ASMR and ASDR. Globally, the ASMR showed a consistent downward trend, with the most significant decline occurring between 2003 and 2007, where the APC reached −3.50%. After that, the decline continued, but at a slower pace. During this period, the overall AAPC of ASMR was −1.80%. In contrast, the ASDR attributable to IS also exhibited a similar declining trend. The steepest decline occurred between 2004 and 2007, with an APC of −3.48%. From 1990 to 2021, the AAPC in global ASDR was similarly negative, at −1.47%.

In China, the trends of ASMR and ASDR attributable to high LDL-C for IS exhibited greater variability across different time periods. From 1998 to 2004, both the ASMR and ASDR rates significantly increased, with APC values of 2.90% and 2.09%, respectively. Subsequently, from 2004 to 2007, there was a sharp decline, with APCs of −4.58% and −3.64%, respectively. From 2007 to 2010, the rates of change slowed again, with APC values of 0.39% and 0.23%. After 2010, there was a continuous decline at varying rates. From 1990 to 2021, the overall AAPC for ASMR in China was −0.42%, and similarly, the AAPC for ASDR was −0.46%.

### 3.5 ASMR and ASDR attributable to high LDL-C for IS in 2021

**Figure 4:**
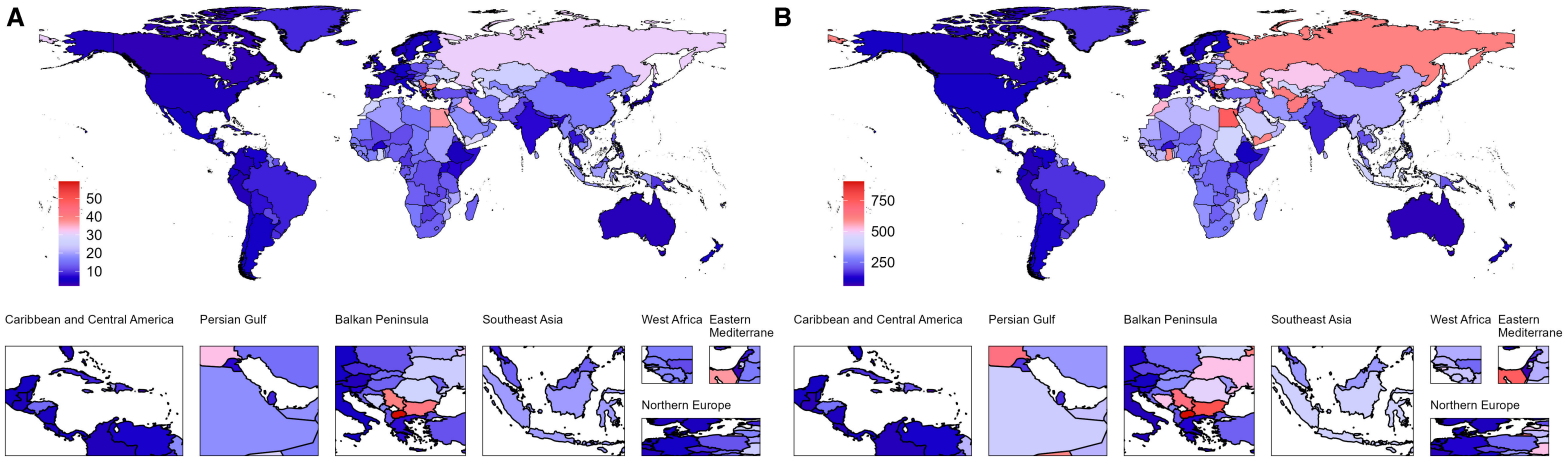
Age-standardized mortality (ASMR) and DALY (ASDR) rates due to IS caused by high LDL-C Globally in 2021. **(A)** ASMR of IS attributable to high LDL-C Globally in 2021. **(B)** ASDR of IS attributable to high LDL-C Globally in 2021.

Figure 4 illustrates the global distribution of mortality and DALYs attributable to high LDL-C. Figure 4A highlights that the burden of mortality was particularly severe in African countries. In countries such as Ethiopia, Kenya, Madagascar, Mauritius, and Malawi, the mortality rates exceed 30 per 100,000 population, with Malawi having the highest rate at 59.37 per 100,000 (95% UI: 4.14–26.59). In China, the ASMR was 18.99 per 100,000 (95% UI: 6.17–34.00).

Figure 4B presents the global distribution of ASDR attributable to high LDL-C. The burden was particularly pronounced in Northeastern and Southeastern Europe as well as parts of the Western Pacific region. In Europe, Lithuania, the Republic of San Marino, and the former Yugoslav Republic of Macedonia reported rates exceeding 745 per 100,000 population, with Lithuania recording the highest rate at 1,167.16 per 100,000 (95% UI: 127.10–559.27). In the Northern Mariana Islands, part of the Western Pacific, the ASDR was 745.99 per 100,000 (95% UI: 284.45–1,565.26). In China, the ASDR was 37.48 per 100,000 (95% UI: 69.17–331.74).

### 3.6 Predictions of the burden of IS attributable to high LDL-C in China and globally

**Figure 5:**
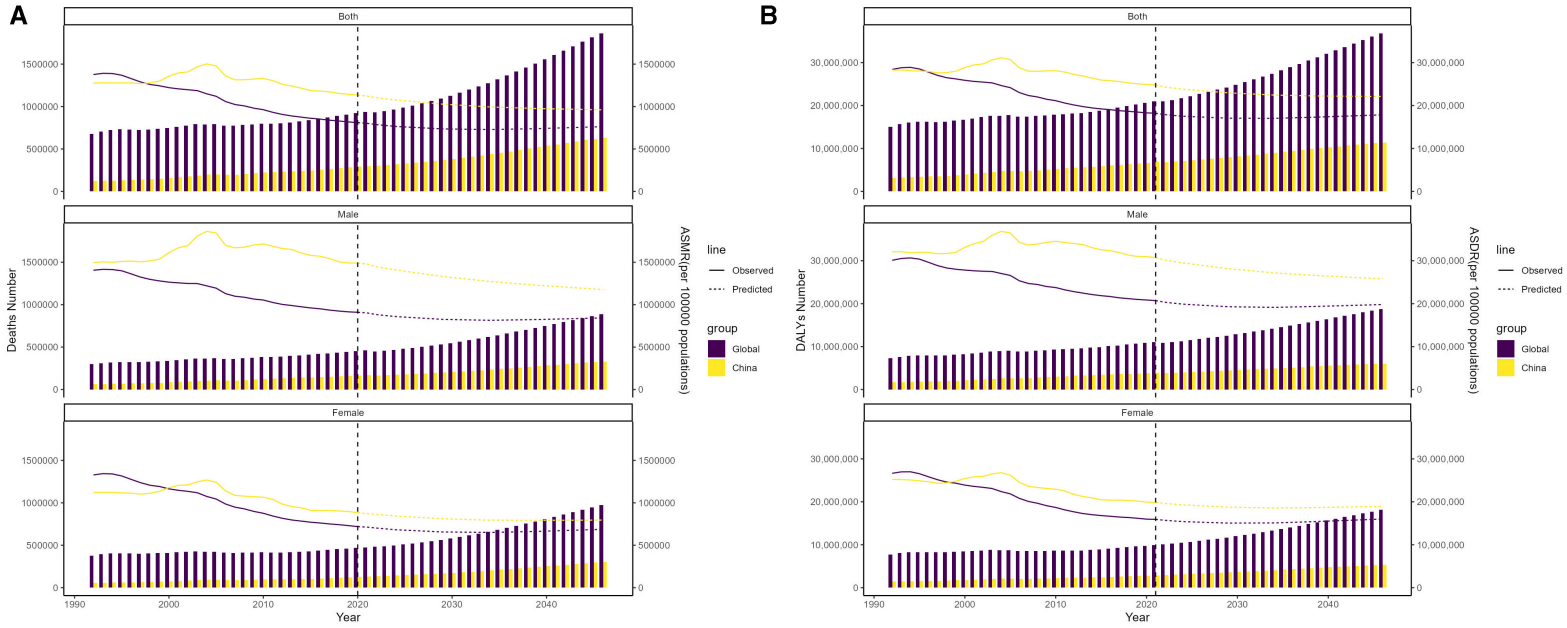
Projected trends in deaths and DALYs due to high LDL-C-related IS from 2022 to 2046 globally and in China. **(A)** Predictions of total, male, and female deaths and ASMR in global and China. **(B)** Predictions of total, male, and female DALYs and ASDR in global and China.

Based on Figure 5A, it is predicted that by 2046, the global number of deaths due to IS caused by high LDL-C will reach 1,863,524, with ASMR of 10.82 per 100,000 people. Among them, the number of male deaths is expected to be 889,875, with an ASMR of 11.97; the number of female deaths is expected to be 973,648, with an ASMR of 9.76. In China, the number of deaths from IS caused by high LDL-C is predicted to reach 629,880 by 2046, with the ASMR slightly decreasing to 13.62 per 100,000 people. The number of male deaths is expected to be 327,183, with an ASMR of 16.70; for females, the number is expected to be 302,698, with an ASMR of 11.30.

As shown in Figure 5B, the global number of DALYs due to IS caused by high LDL-C is predicted to reach 36,833,443 by 2046, with an ASDR of 241.50 per 100,000 people. The DALYs for males are expected to be 18,731,942, with an ASDR of 268.08; for females, the DALYs are expected to be 18,101,501, with an ASDR of 216.50. In China, the DALYs in 2046 are expected to reach 11,376,410, with an ASDR of 299.34. For males, the DALYs are expected to be 6,046,436, with an ASDR of 349.15; for females, the DALYs are expected to be 5,329,974, with an ASDR of 256.46.

## 4 Discussion

Between 1990 and 2021, despite increases in both deaths and DALYs due to high LDL-C levels contributing to IS both globally and in China, ASMR and ASDR have overall decreased. This suggests that the rising burden is primarily driven by population aging and growth (17). Compared to global trends, China has experienced a more pronounced increase in absolute deaths and DALYs, indicating a heavier disease burden. However, the decrease in ASMR and ASDR in China has been smaller than the global average, reflecting greater challenges in mitigating the impact of high LDL-C on IS. This pattern mirrors global cardiovascular disease trends (30). From 1990 to 2021, China's population grew from 1.16 billion to 1.41 billion, with the elderly population (aged 65 and older) surging from 54 million to 191 million. Furthermore, the average life expectancy for Chinese citizens rose from 68.60 years in 1990 to 77.30 years in 2019.

In 2021, both global and Chinese mortality and DALY rates for IS due to high LDL-C exhibited age- and sex-specific patterns. Research indicates that both mortality and DALY rates for IS increase with age, with men generally experiencing higher rates than women. This finding aligns with previous studies (19). In China, male mortality and DALY rates remain higher than those of females up to the age of 84, after which a reversal in the sex difference is observed (19). Additionally, another study highlighted that LDL-C levels rise with age, with distinct trends observed between men and women (31). Collectively, these studies suggest that men face a higher risk of cardiovascular diseases, possibly due to biological, behavioral, and lifestyle factors (32). As global population aging accelerates and lifestyles continue to evolve, the burden of high LDL-C has shown diverse trends across regions and populations (17), underscoring the need for gender-specific prevention and control strategies.

Joint regression analysis further supports the observed trends. Globally, the ASMR for IS attributable to high LDL-C exhibited a consistent decline from 1990 to 2021, with the most significant drop occurring between 2003 and 2007. This trend may be associated with the widespread implementation of lipid-lowering treatment strategies globally during that time (17). In contrast, China's trends were more variable, with significant increases in ASMR and ASDR from 1998 to 2004, a period coinciding with rapid dietary shifts toward animal fat consumption (33) and urban-rural disparities in acute stroke care access (34). The subsequent sharp decline between 2004 and 2007 (30) likely reflects targeted public health interventions, including the rollout of basic lipid screening in urban centers (35). Afterward, the rate of decline slowed, mirroring China's slower adoption of intensive lipid management compared to high-income countries (36). This lag may be attributed to persistent gaps in physician awareness of LDL-C targets (only 38% adherence to guidelines) and urbanization-driven lifestyle risks (e.g., reduced physical activity and processed food intake) (37).

Our projections indicate that while the global ASMR is expected to stabilize by 2046, the total number of deaths will rise, particularly among women. This emphasizes the ongoing need to manage high LDL-C levels and underscores the importance of gender-specific interventions. In China, the ASMR is projected to stay above the global average, signaling a greater burden and the need for targeted public health initiatives and clinical interventions. Additionally, the projected DALYs underscore the long-term health impact of high LDL-C, with men facing higher DALYs, highlighting the need for sex-specific strategies. These projections stress the urgent need for effective cholesterol management policies and population-level interventions.

Our study extends previous research by offering broader temporal and geographic coverage. Compared to earlier research (19), it spans over three decades (1990–2021), incorporates global and national trends, and applies predictive modeling through 2046. It provides a forward-looking perspective on the prevention and control of IS both globally and within China. This study also offers a detailed analysis of gender and age group disparities, showing that the burden of IS in China's elderly population exceeds the global average, with significant gender differences. These insights are crucial for designing targeted prevention strategies. Unlike the study by Zhang et al. (18), this study not only examines trends on a global scale but also provides an in-depth analysis of data specific to China. It uncovers the unique challenges China faces in addressing IS due to high LDL-C, such as the slower decline in ASMR and ASDR compared to the global average. This suggests that China is encountering greater challenges in reducing the impact of high LDL-C on IS and therefore requires more focused and targeted preventive measures.

However, several limitations should be acknowledged. Firstly, the GBD 2021 data do not classify IS subtypes according to specific systems, such as the TOAST or CISS classification, limiting the ability to investigate the underlying causes of IS (5). Secondly, the focus on high LDL-C as a singular risk factor for IS may overlook the complex interactions with other factors, such as hypertension, diabetes, and smoking. Notably, potential synergistic effects among high LDL-C, hypertension, and diabetes may amplify IS risk beyond individual contributions, possibly underestimating the value of integrated prevention strategies (38). The study mainly aggregates data at the national level, potentially masking regional disparities (4). For example, China's diverse healthcare infrastructure and socioeconomic conditions across provinces may lead to substantial variations in IS burden. Similarly, global variations in healthcare systems, public health policies, and cultural contexts may influence the geographic distribution of disease burden (39). Thirdly, while the Nordpred model provides robust long-term forecasting based on historical trends, its projections do not account for potential impacts of emerging therapeutic modalities (particularly novel lipid-lowering agents like PCSK9 inhibitors, bempedoic acid, and siRNA-based therapies) or evolving health policies (such as expanded LDL-C screening programs or combination therapy guidelines) (40). Therefore, the predictive results should be interpreted with caution, as they might not fully reflect real-time dynamics. Furthermore, our use of the GBD LDL-C threshold (>1.30 mmol/L), while enabling global comparisons, differs from China's 2023 guidelines (<1.8 mmol/L for high-risk patients) (41). This may underestimate burden in Chinese high-risk populations, as some GBD-classified “moderate risk” individuals would qualify for intensive therapy locally (42). While valuable for international standardization, these results should be interpreted alongside local guidelines for clinical application.

Future studies should clarify how population growth, aging, and evolving epidemiological patterns contribute to the IS burden associated with high LDL-C. Robust decomposition analyses are needed to quantify the individual and combined effects of these factors (43). Additionally, incorporating individual-level data—such as lifestyle and genetic variables—will improve the accuracy of burden assessments (44). Exploring the interactions between LDL-C and other metabolic risk factors, including hypertension and diabetes, will also provide deeper insight into disease etiology (45). Regional analyses with finer granularity are essential to identify geographic disparities and support targeted, equitable interventions. Finally, integrating diverse predictive models that account for sociocultural and policy-related variables can enhance the relevance and precision of future projections.

## 5 Conclusion

This study provides a comprehensive comparative analysis of the global and Chinese burden of IS attributable to elevated LDL-C from 1990 to 2021, and offers projections of future trends. Despite a rise in the absolute numbers of deaths and DALYs, primarily driven by population growth and aging, the ASMR and ASDR have generally shown a declining trend. In comparison to the global average, China faces more significant challenges in reducing the IS burden attributed to high LDL-C levels. This burden is particularly pronounced among older adults and men, highlighting the urgent need for targeted interventions. Projections suggest a continued rise in the burden of IS, underscoring the need for more effective, gender- and age-specific prevention and intervention strategies to address this growing public health challenge globally and in China.

## Data Availability

The original contributions presented in the study are included in the article/Supplementary material, further inquiries can be directed to the corresponding authors.

## References

[1] Walter K. What is acute ischemic stroke? JAMA. (2022) 327:885. 10.1001/jama.2022.1420 35230392

[2] Wang Q Yang F Duo K Liu Y Yu J Wu Q. The role of necroptosis in cerebral ischemic stroke. Mol Neurobiol. (2024) 61:3882–98. 10.1007/s12035-023-03728-7 38038880

[3] Collaborators GBDSRF. Global, regional, and national burden of stroke and its risk factors, 1990-2021: a systematic analysis for the global burden of disease study 2021. Lancet Neurol. (2024) 23:973–1003. 10.1016/S1474-4422(24)00369-7 39304265 PMC12254192

[4] Hou S Zhang Y Xia Y Liu Y Deng X Wang W. Global, regional, and national epidemiology of ischemic stroke from 1990 to 2021. Eur J Neurol. (2024) 31:e16481. 10.1111/ene.16481 39290044 PMC11555022

[5] Li XY Kong XM Yang CH Cheng ZF Lv JJ Guo H. Global, regional, and national burden of ischemic stroke, 1990-2021: an analysis of data from the global burden of disease study 2021. EClinicalMedicine. (2024) 75:102758. 10.1016/j.eclinm.2024.102758 39157811 PMC11327951

[6] Tu WJ Zhao Z Yin P Cao L Zeng J Chen H. Estimated burden of stroke in China in 2020. JAMA Network Open. (2023) 6:e231455. 10.1001/jamanetworkopen.2023.1455 36862407 PMC9982699

[7] Ji C Ge X Zhang J Tong H. The stroke burden in China and its long-term trends: insights from the global burden of disease (GBD) study 1990-2021. Nutr Metab Cardiovasc Dis (2025):103848. 10.1016/j.numecd.2025.103848 39948019

[8] Rochmah TN Rahmawati IT Dahlui M Budiarto W Bilqis N. Economic burden of stroke disease: a systematic review. Int J Environ Res Public Health. (2021) 18:7552. 10.3390/ijerph18147552 34299999 PMC8307880

[9] Amarenco P Kim JS Labreuche J Charles H Abtan J Bejot Y. A comparison of two ldl cholesterol targets after ischemic stroke. N Engl J Med. (2020) 382:9. 10.1056/NEJMc2001195 31738483

[10] Amarenco P Kim JS Labreuche J Charles H Giroud M Lee BC. Benefit of targeting a LDL (low-density lipoprotein) cholesterol <70 Mg/Dl during 5 years after ischemic stroke. Stroke. (2020) 51:1231–9. 10.1161/STROKEAHA.119.028718 32078484

[11] Krahenbuhl S Pavik-Mezzour I von Eckardstein A. Unmet needs in LDL-C lowering: when statins won't do! Drugs. (2016) 76:1175–90. 10.1007/s40265-016-0613-0 27456066 PMC4974266

[12] Huang LZ Zhu HB. Novel LDL-oriented pharmacotherapeutical strategies. Pharmacol Res. (2012) 65:402–10. 10.1016/j.phrs.2012.01.007 22306845

[13] Chen ZM Gu HQ Mo JL Yang KX Jiang YY Yang X. U-shaped association between low-density lipoprotein cholesterol levels and risk of all-cause mortality mediated by post-stroke infection in acute ischemic stroke. Sci Bull. (2023) 68:1327–35. 10.1016/j.scib.2023.05.028 37270342

[14] Joint Committee on the Chinese Guidelines for Lipid Management. Chinese guideline for lipid management (primary care version 2024). J Clin Cardiol. (2024) 40:249–56. 10.13201/j.issn.1001-1439.2024.04.001 38548600

[15] Duan X Zhang M Sun X Lin Y Peng W. A lasso-derived model for the prediction of nonattainment of target LDL-C reduction with Pcsk9 inhibitors in patients with atherosclerotic cardiovascular disease. Lipids Health Dis. (2025) 24:65. 10.1186/s12944-025-02488-8 39985079 PMC11846231

[16] Ma Q Li R Wang L Yin P Wang Y Yan C. Temporal trend and attributable risk factors of stroke burden in China, 1990-2019: an analysis for the global burden of disease study 2019. Lancet Public Health. (2021) 6:e897–906. 10.1016/S2468-2667(21)00228-0 34838196 PMC9047702

[17] Du H Shi Q Song P Pan XF Yang X Chen L. Global burden attributable to high low-density lipoprotein-cholesterol from 1990 to 2019. Front Cardiovasc Med. (2022) 9:903126. 10.3389/fcvm.2022.903126 35757342 PMC9218272

[18] Zhang J Zhu S Liu C Hu Y Yang A Zhang Y. Global, regional and national burden of ischemic stroke attributed to high low-density lipoprotein cholesterol, 1990-2019:a decomposition analysis and age-period-cohort analysis. J Cereb Blood Flow Metab. (2024) 44:527–41. 10.1177/0271678X231211448 37891501 PMC10981397

[19] Deng Z Li H Wang J. Temporal trends of the burden of ischemic stroke attributable to high low-density lipoprotein cholesterol in China from 1999 to 2019. BMC Public Health. (2024) 24:3003. 10.1186/s12889-024-20461-5 39478553 PMC11523588

[20] Collaborators GBDCoD. Global burden of 288 causes of death and life expectancy decomposition in 204 countries and territories and 811 subnational locations, 1990-2021: a systematic analysis for the global burden of disease study 2021. Lancet. (2024) 403:2100–32. 10.1016/S0140-6736(24)00367-2 38582094 PMC11126520

[21] Collaborators GBDNSD. Global, regional, and national burden of disorders affecting the nervous system, 1990-2021: a systematic analysis for the global burden of disease study 2021. Lancet Neurol. (2024) 23:344–81. 10.1016/S1474-4422(24)00038-3 38493795 PMC10949203

[22] Stevens GA Alkema L Black RE Boerma JT Collins GS Ezzati M. Guidelines for accurate and transparent health estimates reporting: the gather statement. Lancet. (2016) 388:e19–23. 10.1016/S0140-6736(16)30388-9 27371184

[23] Collaborators GBDRF. Global burden of 87 risk factors in 204 countries and territories, 1990-2019: a systematic analysis for the global burden of disease study 2019. Lancet. (2020) 396:1223–49. 10.1016/S0140-6736(20)30752-2 33069327 PMC7566194

[24] Aho K Harmsen P Hatano S Marquardsen J Smirnov VE Strasser T. Cerebrovascular disease in the community: results of a who collaborative study. Bull World Health Organ. (1980) 58:113–30. 6966542 PMC2395897

[25] Kim HJ Chen HS Byrne J Wheeler B Feuer EJ. Twenty years since joinpoint 10: two major enhancements, their justification, and impact statistics in medicine. Stat Med. (2022) 41:3102–30. 10.1002/sim.9407 35522060

[26] Qiu H Cao S Xu R. Cancer incidence, mortality, and burden in china: a time-trend analysis and comparison with the United States and United Kingdom based on the global epidemiological data released in 2020. Cancer Commun. (2021) 41:1037–48. 10.1002/cac2.12197 34288593 PMC8504144

[27] Arnold M Park JY Camargo MC Lunet N Forman D Soerjomataram I. Is gastric cancer becoming a rare disease? a global assessment of predicted incidence trends to 2035. Gut. (2020) 69:823–9. 10.1136/gutjnl-2019-320234 32001553 PMC8520492

[28] Luo G Zhang Y Etxeberria J Arnold M Cai X Hao Y. Projections of lung cancer incidence by 2035 in 40 countries worldwide: population-based study. JMIR Public Health Surveill. (2023) 9:e43651. 10.2196/43651 36800235 PMC9984998

[29] Liu X Wu B Lai Y Zhang X Li H Qu F. Temporal trends in the burden of diabetes and its risk factors across the western pacific region between 1990 and 2044: a systematic analysis of the global burden of disease study 2019. Diabetes Metab Res Rev. (2025) 41:e70036. 10.1002/dmrr.70036 39983069 PMC11845174

[30] Zheng J Wang J Zhang Y Xia J Guo H Hu H. The global burden of diseases attributed to high low-density lipoprotein cholesterol from 1990 to 2019. Front Public Health. (2022) 10:891929. 10.3389/fpubh.2022.891929 36051998 PMC9424500

[31] Zhang P Su Q Ye X Guan P Chen C Hang Y. Trends in Ldl-C and Non-Hdl-C levels with age. Aging Dis. (2020) 11:1046–57. 10.14336/AD.2019.1025 33014521 PMC7505266

[32] Rosenberg K. High LDL-C, non-HDL-C levels associated with higher CVD death risk, even in those at low CVD risk. Am J Nurs. (2018) 118:67. 10.1097/01.NAJ.0000549699.94445.68 30461501

[33] Chen X Zhong F Li J. The burden of cardiovascular disease attributable to dietary risk factors in China, 1990-2021. Sci Rep. (2025) 15:25641. 10.1038/s41598-025-11645-z 40664912 PMC12264015

[34] Hammond G Luke AA Elson L Towfighi A Joynt Maddox KE. Urban-rural inequities in acute stroke care and in-hospital mortality. Stroke. (2020) 51:2131–8. 10.1161/STROKEAHA.120.029318 32833593

[35] Zhou F Zhou J Wang W Zhang XJ Ji YX Zhang P. Unexpected rapid increase in the burden of NAFLD in China from 2008 to 2018: a systematic review and meta-analysis. Hepatology. (2019) 70:1119–33. 10.1002/hep.30702 31070259

[36] Collaborators GBDS. Global, regional, and national burden of stroke and its risk factors, 1990-2019: a systematic analysis for the global burden of disease study 2019. Lancet Neurol. (2021) 20:795–820. 10.1016/S1474-4422(21)00252-0 34487721 PMC8443449

[37] Banach M Reiner Z Surma S Bajraktari G Bielecka-Dabrowa A Bunc M. 2024 recommendations on the optimal use of lipid-lowering therapy in established atherosclerotic cardiovascular disease and following acute coronary syndromes: a position paper of the international lipid expert panel (ILEP). Drugs. (2024) 84:1541–77. 10.1007/s40265-024-02105-5 39497020 PMC11652584

[38] Lewis GF Hegele RA. Effective, disease-modifying, clinical approaches to patients with mild-to-moderate hypertriglyceridaemia. Lancet Diabetes Endocrinol. (2022) 10:142–8. 10.1016/S2213-8587(21)00284-9 34922644

[39] Diseases GBD Injuries C. Global burden of 369 diseases and injuries in 204 countries and territories, 1990-2019: a systematic analysis for the global burden of disease study 2019. Lancet. (2020) 396:1204–22. 10.1016/S0140-6736(20)30925-9 33069326 PMC7567026

[40] Pan H Tang Y Zhu H Sun Y Chi P Huang Y. Global burden, trends, and risk factors of early-onset and late-onset colorectal cancer from 1990 to 2021, with projections to 2040: a population-based study. BMC Gastroenterol. (2025) 25:486. 10.1186/s12876-025-04086-5 40597636 PMC12211933

[41] Liu BQ Yang C Wei HY Yu ZX. Global, regional, and national burden of ischemic heart disease attributable to metabolic risks: a systematic analysis of global burden of disease 2021. J Geriatr Cardiol. (2025) 22:361–80. 10.26599/1671-5411.2025.03.009 40351395 PMC12059566

[42] Tang J Zhou G Shi S Lu Y Cheng L Xiang J. Systematic analysis of the burden of ischemic stroke attributable to high LDL-C from 1990 to 2021. Front Neurol. (2025) 16:1547714. 10.3389/fneur.2025.1547714 40255889 PMC12005992

[43] Li J Chan NB Xue J Tsoi KKF. Time series models show comparable projection performance with joinpoint regression: a comparison using historical cancer data from world health organization. Front Public Health. (2022) 10:1003162. 10.3389/fpubh.2022.1003162 36311591 PMC9614249

[44] Yang B Ma X Yang L Bian G Qiao B Lu H. Trends and prospects of low-density lipoprotein cholesterol in stroke: a bibliometric analysis. Cureus. (2024) 16:e69492. 10.7759/cureus.69492 39421126 PMC11485023

[45] Banach M Shekoohi N Mikhailidis DP Lip GYH Hernandez AV Mazidi M. Relationship between low-density lipoprotein cholesterol, lipid-lowering agents and risk of stroke: a meta-analysis of observational studies (N = 355,591) and randomized controlled trials (N = 165,988). Arch Med Sci. (2022) 18:912–29. 10.5114/aoms/145970 35832716 PMC9266957

