# Supplementary material for: Comparing burden of ischemic stroke caused by high low-density lipoprotein cholesterol in global and China: trends and projections
Source: Front Neurol. 2025 Aug 13;16:1622361. doi: 10.3389/fneur.2025.1622361 (PMC12380887; doi:10.3389/fneur.2025.1622361)
Supplement: Supplementary file 1 [file Table_1.doc]

Supplementary Table 1. Overall Data from the Joinpoint Regression Analysis of ASMR and ASDR due to IS caused by high LDL-C Globally and in China from 1990 to 2021.

| **Group** | **ASMR** | | |  | **ASDR** | | |
| --- | --- | --- | --- | --- | --- | --- | --- |
|  | **Period** | **APC(%)** | **AAPC(%)** |  | **Period** | **APC(%)** | **AAPC(%)** |
| **Global** | **1990-1995** | **-0.44*** | **-1.8** |  | **1990-1995** | **-0.08** | **-1.47** |
|  | **1995-1998** | **-2.95*** |  |  | **1995-1998** | **-2.58*** |  |
|  | **1998-2003** | **-1.08*** |  |  | **1998-2003** | **-1.01*** |  |
|  | **2003-2007** | **-3.50*** |  |  | **2003-2007** | **-3.48*** |  |
|  | **2007-2013** | **-2.47*** |  |  | **2007-2013** | **-2.02*** |  |
|  | **2013-2021** | **-1.29*** |  |  | **2013-2021** | **-0.94*** |  |
| **China** | **1990-1998** | **-0.08** | **-0.42** |  | **1990-1998** | **-0.40*** | **-0.46** |
|  | **1998-2004** | **2.90*** |  |  | **1998-2004** | **2.09*** |  |
|  | **2004-2007** | **-4.58*** |  |  | **2004-2007** | **-3.64*** |  |
|  | **2007-2010** | **0.39** |  |  | **2007-2010** | **0.23** |  |
|  | **2010-2015** | **-2.18*** |  |  | **2010-2015** | **-1.61*** |  |
|  | **2015-2021** | **-0.95*** |  |  | **2015-2021** | **-0.82*** |  |

Abbreviations: ASMR, Age-Standardized Mortality Rate, ASDR, Age-Standardized Disability-Adjusted Life Year Rate. IS, Ischemic Stroke. LDL-C, Low-density lipoprotein cholesterol. APC, Annual percent change. AAPC, Average annual percent change.
